# Supplementary material for: A theoretical framework for analyzing coupled neuronal networks: Application to the olfactory system
Source: PLoS Comput Biol. 2017 Oct 2;13(10):e1005780. doi: 10.1371/journal.pcbi.1005780 (PMC5638622; doi:10.1371/journal.pcbi.1005780)
Supplement: S1 Text — This file shows the trial-averaged spiking statistics of the experimental data separated by odor identity. Contains Figs. S1-S8 that show the spiking statistics plotted by the four different odors. Contains Table S1 (Average firing rate by odor), Table S2 (Standard deviation of firing rate by odor). (PDF) [file pcbi.1005780.s001.pdf]

Experimental Data Statistics by Odor

Andrea K. Barreiro[#] ; Shree Hari Gautam[‡] ; Woodrow L. Shew[‡] ; Cheng Ly[†]

# Department of Mathematics, Southern Methodist University, Dallas, TX 75275 U.S.A.
‡Department of Physics, University of Arkansas, Fayetteville, AR 72701 U.S.A.
†Department of Statistical Sciences and Operations Research, Virginia Commonwealth University, Richmond, VA 23284 U.S.A.
∗ E-mail: abarreiro@smu.edu ; shgautam@uark.edu; woodrowshew@gmail.com ; CLy@vcu.edu

Table S1: **Average population firing rate (Hz) by odor and activity state.**

Mean across population	All Odors	Odor 1	Odor 2	Odor 3	Odor 4
ν_{OB}^{Sp}	1.97	1.44	1.8	2.62	2.24
ν_{OB}^{Ev}	4.66	4.91	3.28	5.41	5.34
ν_{PC}^{Sp}	0.75	0.56	0.91	0.74	0.79
ν_{PC}^{Ev}	1.45	1.6	1.26	1.7	1.23

Table S2: **Standard deviation of firing rate across the population (Hz) by odor and activity state.**

Std. Dev. across population	All Odors	Odor 1	Odor 2	Odor 3	Odor 4
ν_{OB}^{Sp}	3.28	2.34	3.07	4.32	3.58
ν_{OB}^{Ev}	7.14	7.55	5.55	8	8.04
ν_{PC}^{Sp}	0.93	0.83	1.08	0.96	0.95
ν_{PC}^{Ev}	1.58	2.09	1.45	1.93	1.18

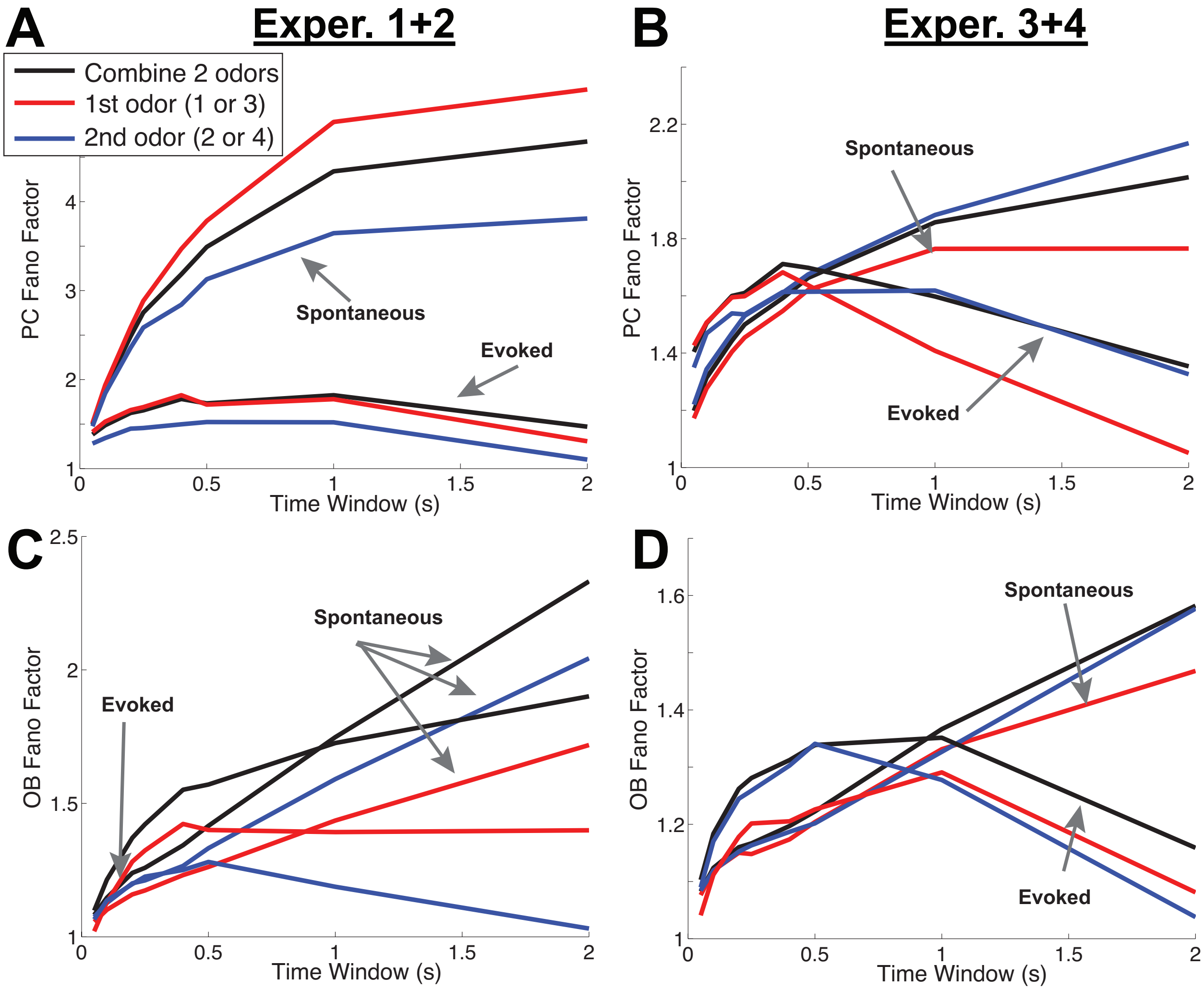

Figure S1: **Experimental statistics by odor and activity state: Fano Factor.** Comparing the **mean** Fano Factor across all simultaneously cells with: i) pairs from the 2 stimuli (black), ii) from the first odor (red), iii) from the second odor (blue). A and B are the PC cells, C and D are the OB cells. The left column A), C) is from `data040515_exp1+2.mat`, and the right column B), D) is from `data040515_exp3+4.mat` The spontaneous and evoked states in groups of 3 curves are denoted by the gray arrows.

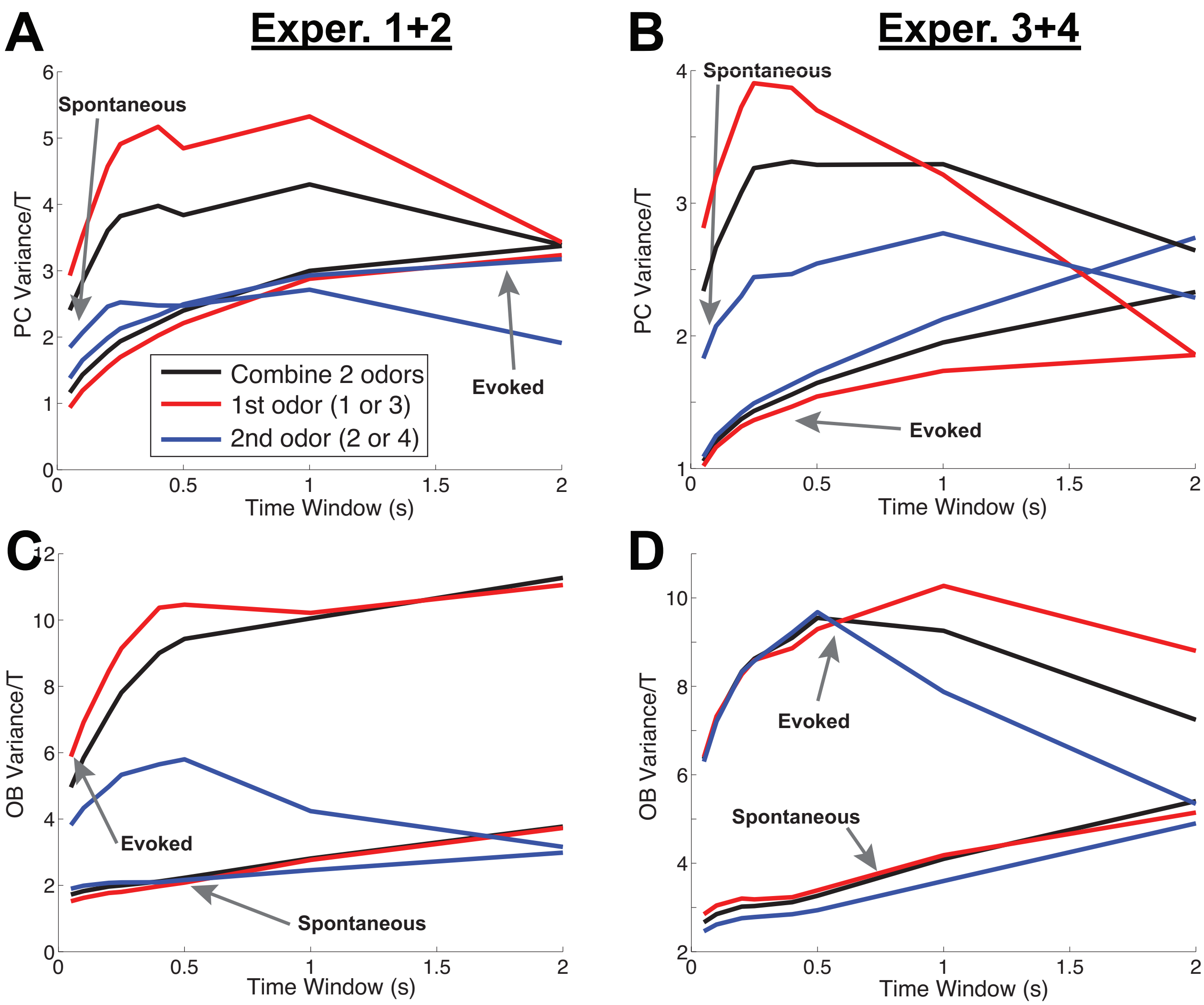

Figure S2: **Experimental statistics by odor and activity state: spike count variance.** Similar to Fig. S1 but comparing the **mean** variance divided by time window across all simultaneously cells with: i) pairs from the 2 stimuli (black), ii) from the first odor (red), iii) from the second odor (blue). A and B are the PC cells, C and D are the OB cells. The left column A), C) is from `data040515_exp1+2.mat`, and the right column B), D) is from `data040515_exp3+4.mat` The spontaneous and evoked states in groups of 3 curves are denoted by the gray arrows.

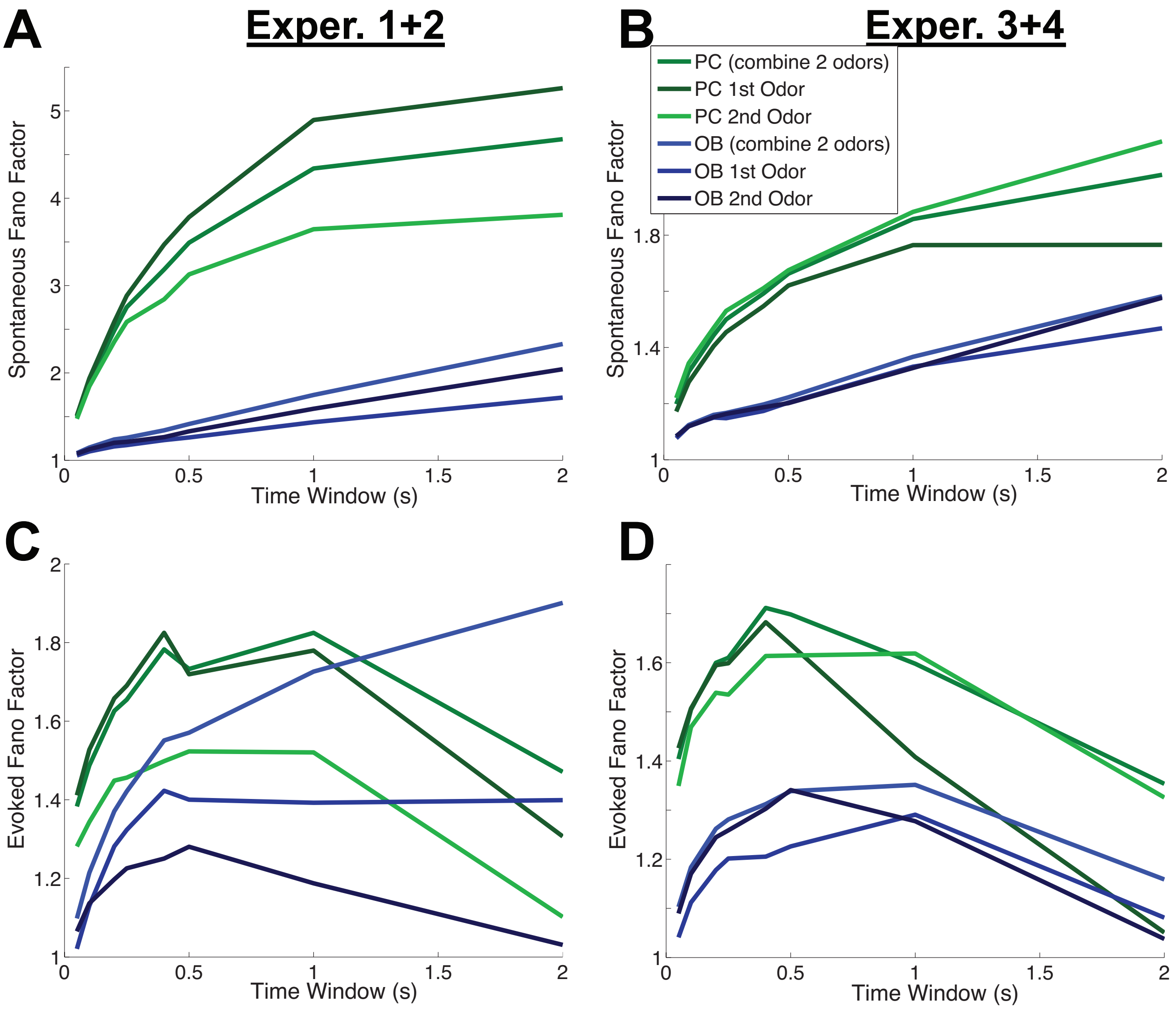

Figure S3: **Experimental statistics by odor and region: Fano Factor.** Comparing the **mean** Fano Factor between recorded PC (3 green curve) and OB (3 blue curves) cells, with: i) pairs from the 2 stimuli , ii) from the first odor, iii) from the second odor (see figure legend for color convention). A and B is for the spontaneous state, C and D is for the evoked state. The left column A), C) is from `data040515_exp1+2.mat`, and the right column B), D) is from `data040515_exp3+4.mat`.

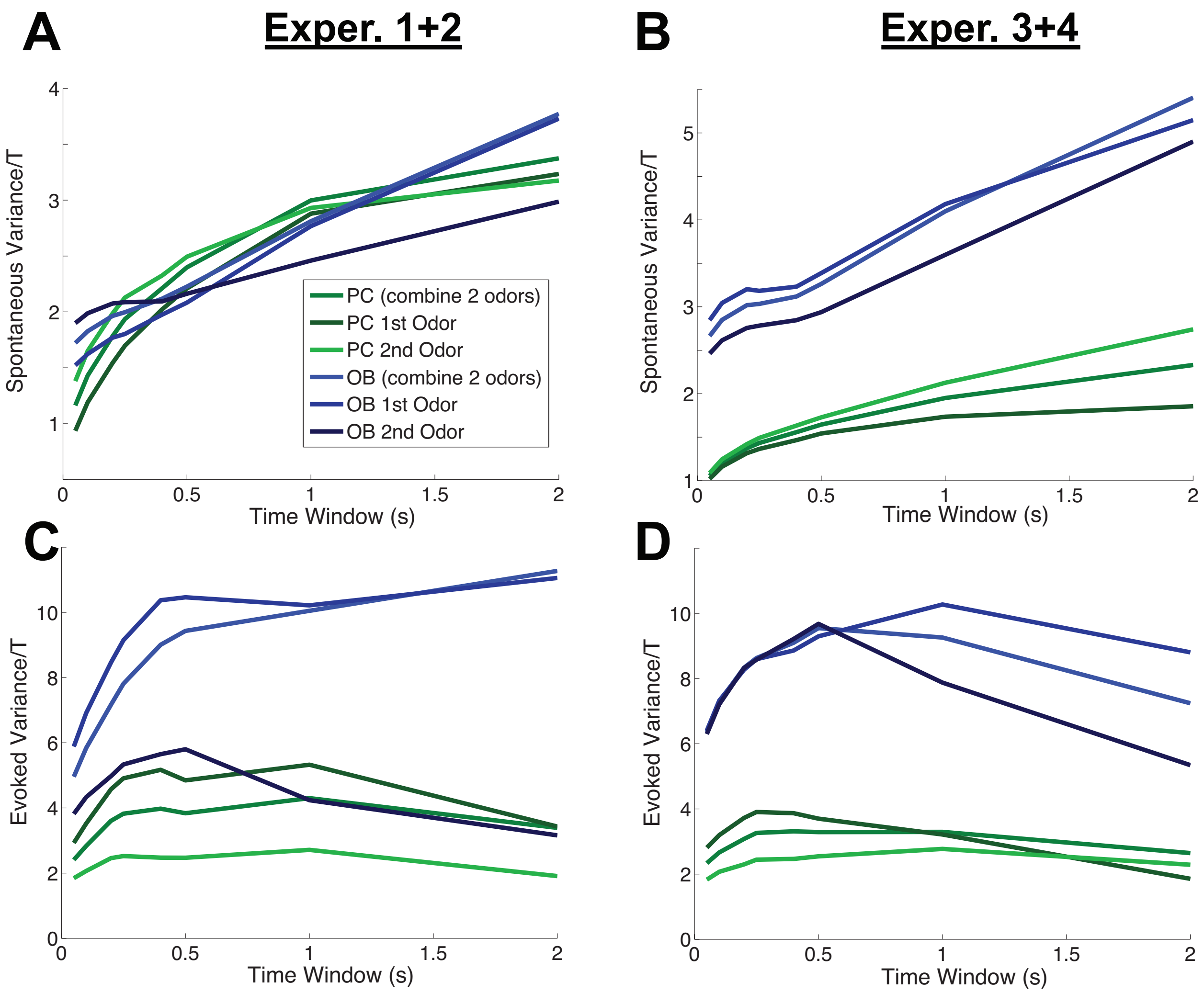

Figure S4: **Experimental statistics by odor and region: spike count variance.** Comparing the **mean** variance divided by time window between recorded PC (3 green curve) and OB (3 blue curves) cells, with: i) pairs from the 2 stimuli , ii) from the first odor, iii) from the second odor (see figure legend for color convention). A and B is for the spontaneous state, C and D is for the evoked state. The left column A), C) is from `data040515_exp1+2.mat`, and the right column B), D) is from `data040515_exp3+4.mat`.

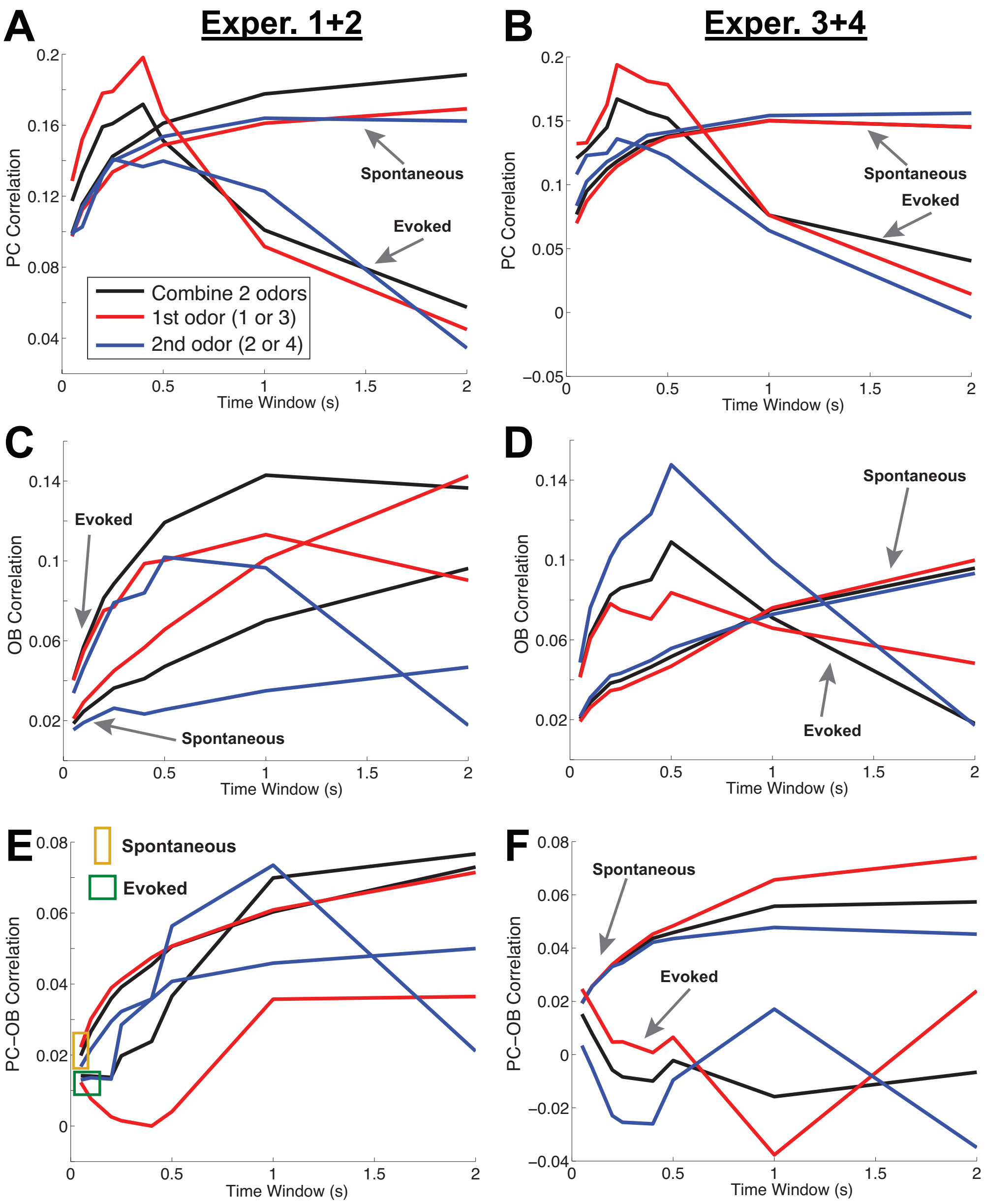

Figure S5: **Experimental statistics by odor and activity state: spike count correlation.** Comparing the **mean** spike count correlation across all simultaneously recorded pairs with: i) pairs from the 2 stimuli (black), ii) from the first odor (red), iii) from the second odor (blue). The left column A), C), E) is from `data040515_exp1+2.mat`, and the right column B), D), F) is from `data040515_exp3+4.mat` The spontaneous and evoked states in groups of 3 curves are denoted by the gray arrows.

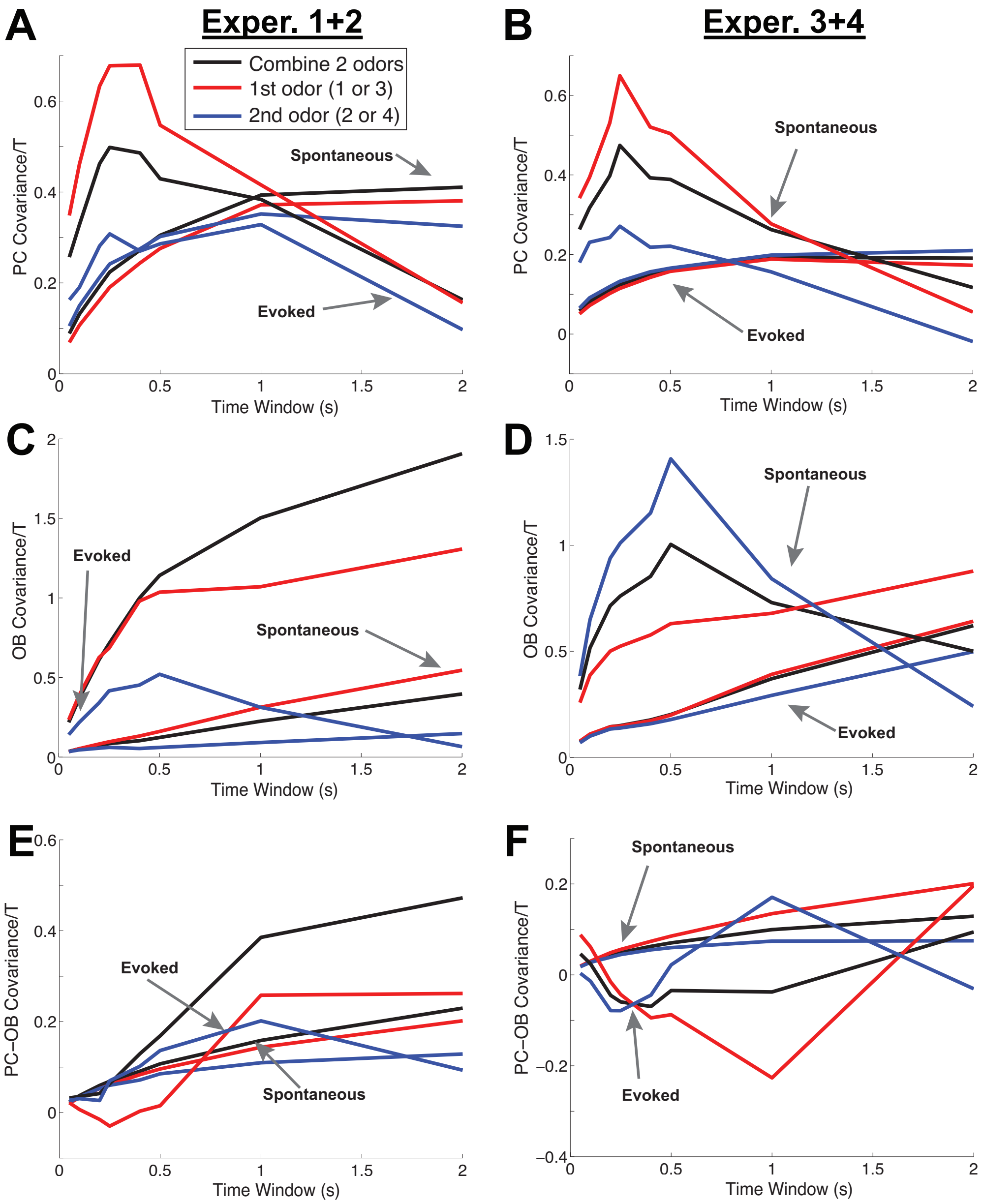

Figure S6: **Experimental statistics by odor and activity state: spike count covariance.** Similar to Fig. S5 but comparing the **mean** spike count covariance divided by time window across all simultaneously recorded pairs with: i) pairs from the 2 stimuli (black), ii) from the first odor (red), iii) from the second odor (blue). The left column A), C), E) is from `data040515_exp1+2.mat`, and the right column B), D), F) is from `data040515_exp3+4.mat` The spontaneous and evoked states in groups of 3 curves are denoted by the gray arrows.

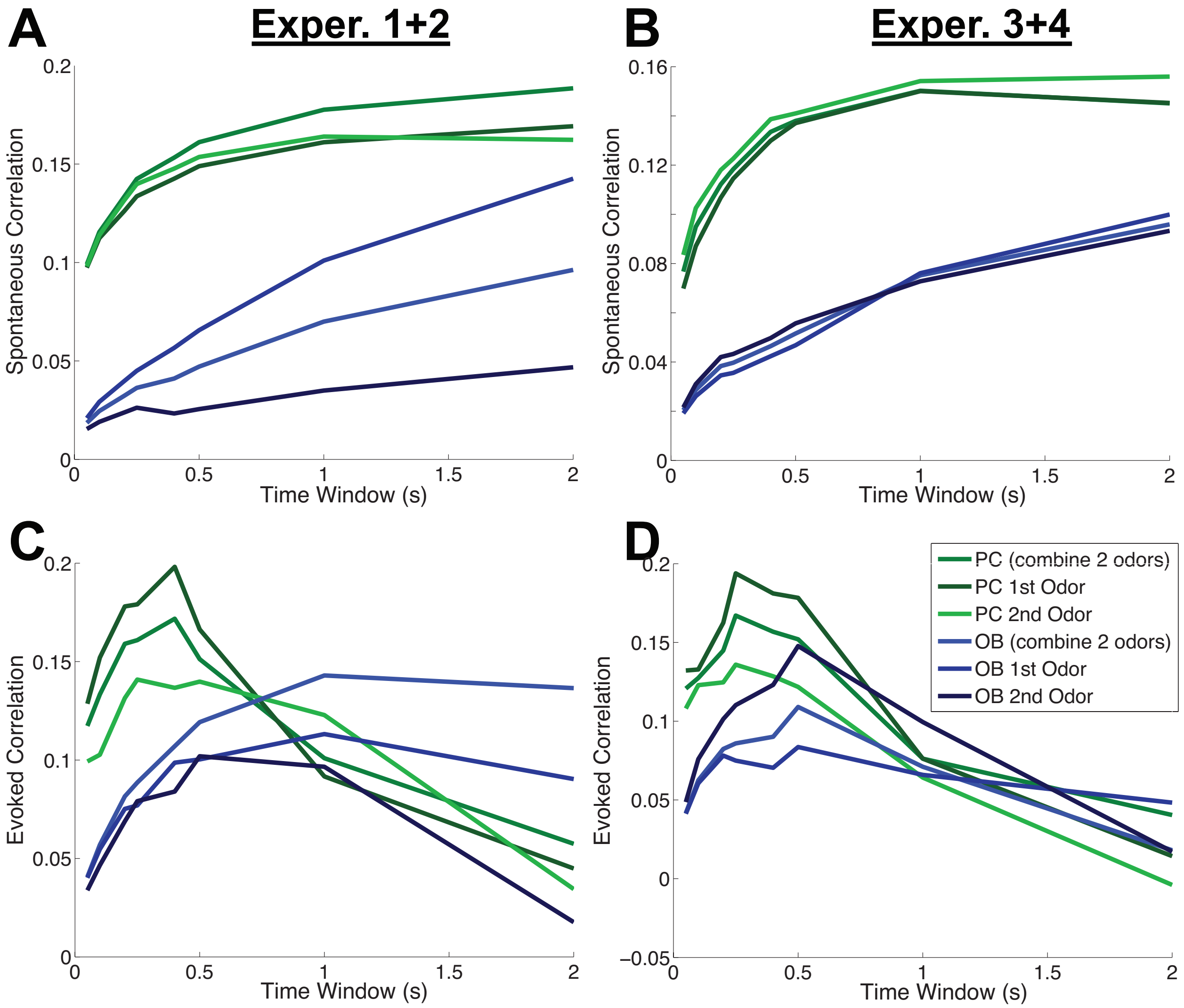

Figure S7: **Experimental statistics by odor and region: spike count correlation.** Comparing the **mean** spike count correlation between all pairs of PC (3 green curve) and OB (3 blue curves) cells, with: i) pairs from the 2 stimuli , ii) from the first odor, iii) from the second odor (see figure legend for color convention). This data constraint was chosen because for larger time windows, it held by odor and experiments. The left column A), C) is from `data040515_exp1+2.mat`, and the right column B), D) is from `data040515_exp3+4.mat`.

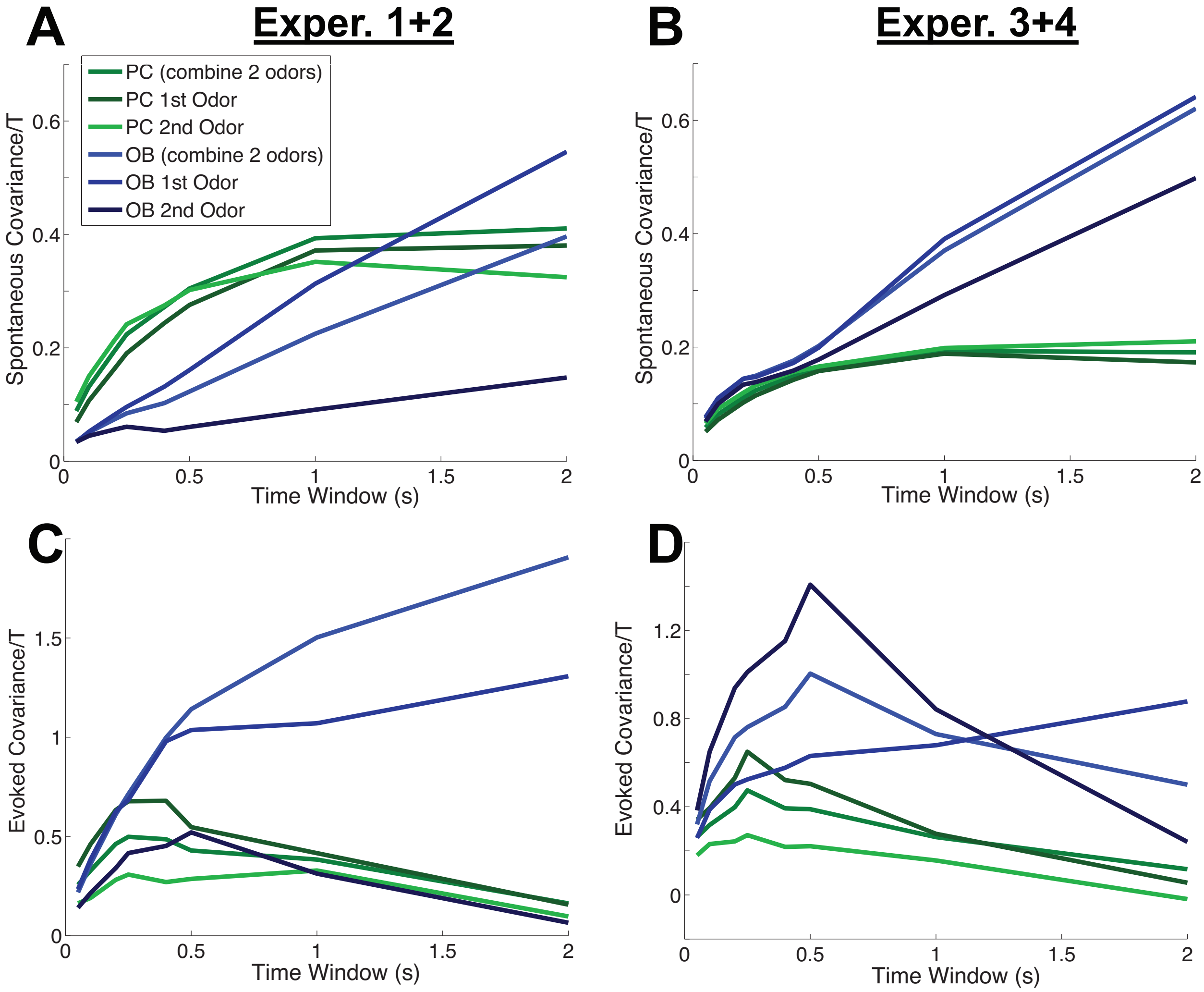

Figure S8: **Experimental statistics by odor and region: spike count covariance.** Comparing the **mean** spike count covariance divided by time window between all pairs of PC (3 green curve) and OB (3 blue curves) cells, with: i) pairs from the 2 stimuli , ii) from the first odor, iii) from the second odor (see figure legend for color convention). The left column A), C), E) is from `data040515_exp1+2.mat`, and the right column B), D), F) is from `data040515_exp3+4.mat`.
