# Supplementary material for: A theoretical framework for analyzing coupled neuronal networks: Application to the olfactory system
Source: PLoS Comput Biol. 2017 Oct 2;13(10):e1005780. doi: 10.1371/journal.pcbi.1005780 (PMC5638622; doi:10.1371/journal.pcbi.1005780)
Supplement: S2 Text — This file contains supplemental figures from modeling and analysis. Contains Figs. S9-S16. S9 Fig: Cross-region correlations are smaller than within-region correlations, S10 Fig: Fast analytic approximation accurately captures statistics of a multi-population firing rate model; S11 Fig: Experimental observations constrain conductance parameters in analytic model; S12 Fig: Analytic approximation results are robust to choice of transfer function; S13 Fig: Mean input to PC must increase in the evoked state for LIF model; S14 Fig: Violating derived relationship gIO < gIP results in statistics that are inconsistent with experimental observations; S15 Fig: Violating derived relationship gEP > gEO results in statistics that are inconsistent with experimental observations; S16 Fig: Violating derived relationship gEP, gIP ≫ gEO, gIO results in statistics that are inconsistent with experimental observations. (PDF) [file pcbi.1005780.s002.pdf]

Supplementary Figures for the Main Modeling

Andrea K. Barreiro[#] ; Shree Hari Gautam[‡] ; Woodrow L. Shew[‡] ; Cheng Ly[†]

# Department of Mathematics, Southern Methodist University, Dallas, TX 75275 U.S.A.
‡Department of Physics, University of Arkansas, Fayetteville, AR 72701 U.S.A.
†Department of Statistical Sciences and Operations Research, Virginia Commonwealth University, Richmond, VA 23284 U.S.A.
∗ E-mail: abarreiro@smu.edu ; shgautam@uark.edu; woodrowshew@gmail.com ; CLy@vcu.edu

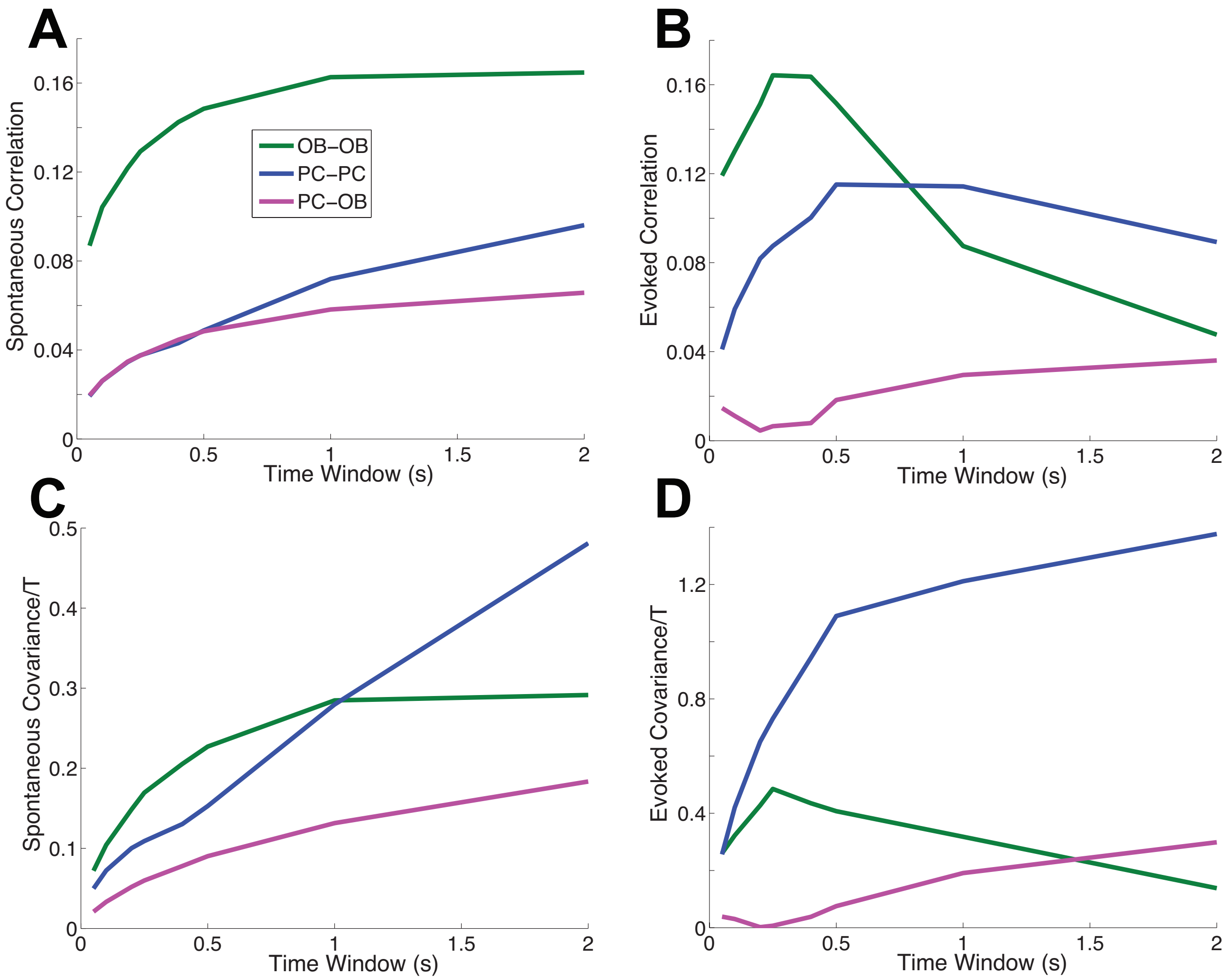

Figure S9: **Cross-region correlations are smaller than within-region correlations.** The experimental data shows that the PC-OB correlation and covariance is small (on average) compared to both OB and PC. A: In the spontaneous state, the (average) Fano Factor of the PC cells is larger than the OB cells. B: In the evoked state, the (average) variance of spike counts of OB cells is larger than the PC cells; here, we have divided by the time window for illustration purposes (which obviously does not change the relationship). In both A and B, there are 73 PC cells and 41 OB cells. C: In the evoked state, the (average) OB covariance is larger than the PC covariance. D: The evoked variance among OB cells is larger than the spontaneous OB variance. In C and D, the covariances were scaled by the time window for illustration purposes, and there were 1298 pairs of PC cells and 406 pairs of OB cells.

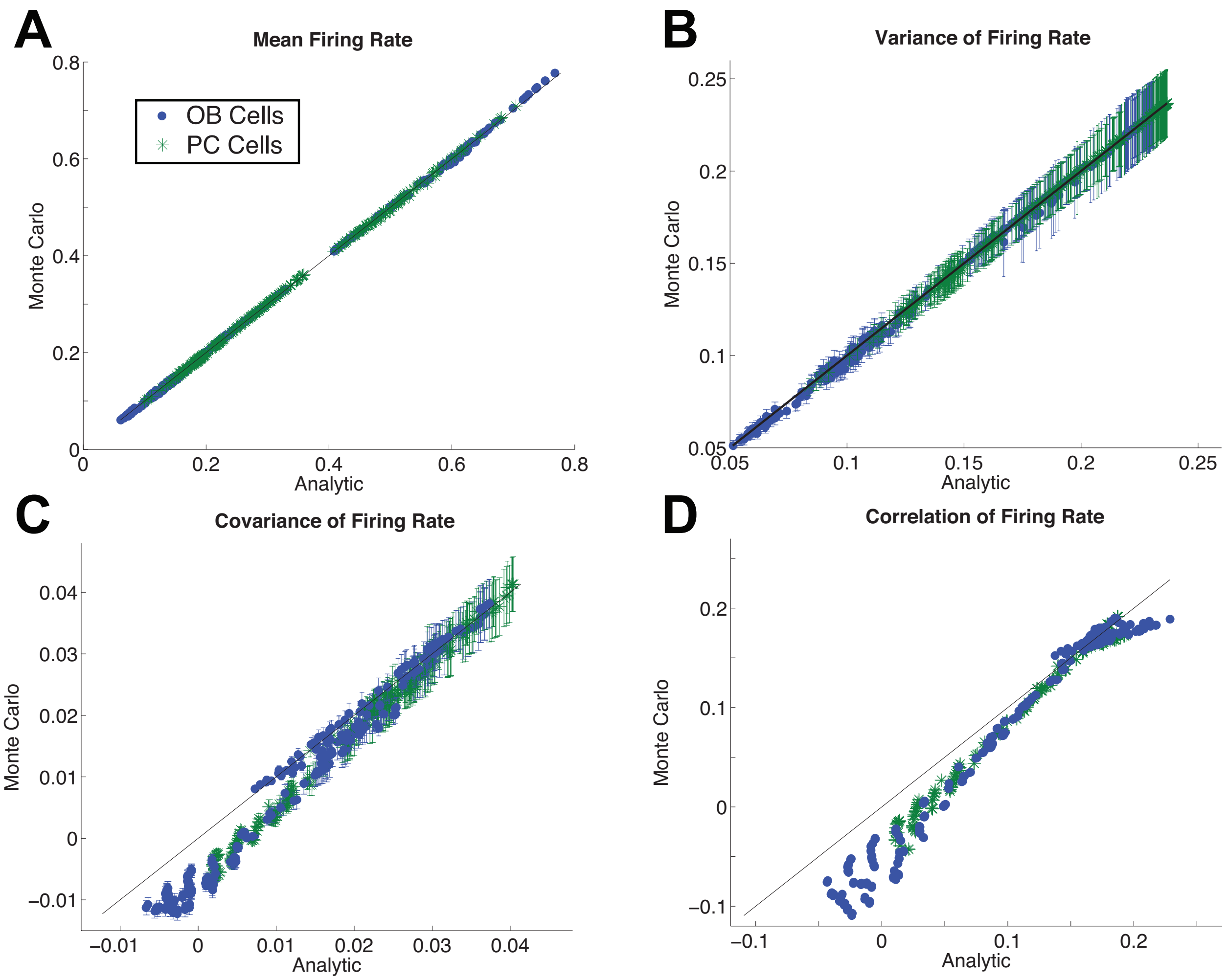

Figure S10: **Fast analytic approximation accurately captures statistics of a multi-population firing rate model.** Comparing the results of the fast analytic approximation to Monte Carlo simulations from 100 randomly selected parameters in the 6 equation rate model: $-2 \leq gIO < 0$, $-2 \leq gIP < 0$, $0 < gEO \leq 2$, $0 < gEP \leq 2$. Comparing 4 important firing rate statistics on a cell by cell basis (i.e., not the average across the population); the statistics for the activity X_j are just as accurate (not shown). A: The mean firing rate $F(X_j)$. B: The variance of the firing rate $Var(F(X_j))$. C: The covariance of the firing rate between OB pairs and PC pairs (we do not focus on OB–PC pairs): $Cov(F(X_j), F(X_k))$. D: The correlation of the firing rate between OB and PC pairs: $\rho = Cov(F(X_j), F(X_k))/\sqrt{Var(F(X_j))Var(F(X_k))}$. The fast analytic approximation is accurate (dots lie on the diagonal line). Error bars are shown in B and C, representing 95% confidence intervals assuming a normal distribution for finite number of realizations, or 1.96 standard deviations above and below the mean.

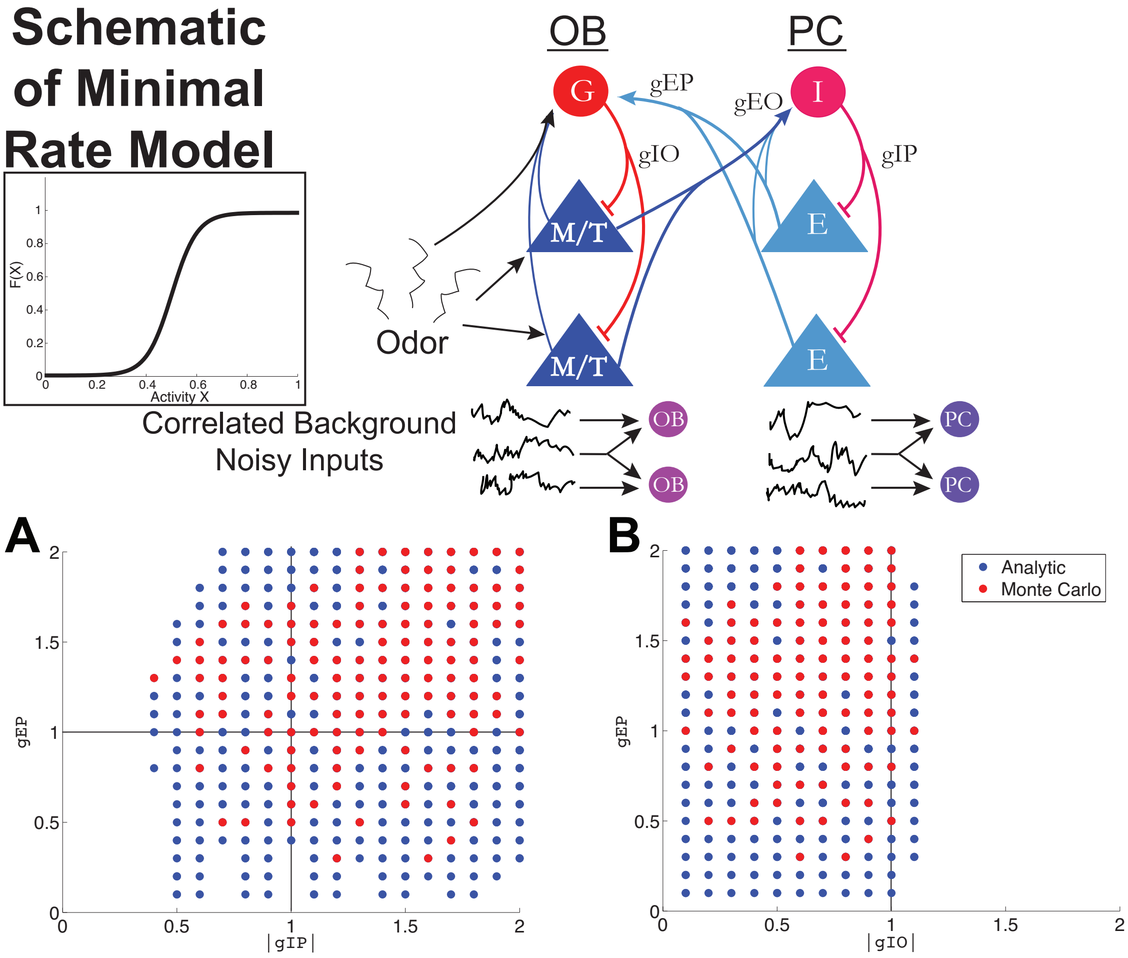

Figure S11: **Experimental observations constrain conductance parameters in analytic model.** The final 2 relationships between the 4 conductance parameters from the fast analytic theory for the rate model not shown in the main text with $F(X) = \frac{1}{2}(1 + \tanh((X - 0.5)/0.1))$. A: Both gEP and $|gIP|$ are relatively large. B: $|gIO|$ is relatively small.

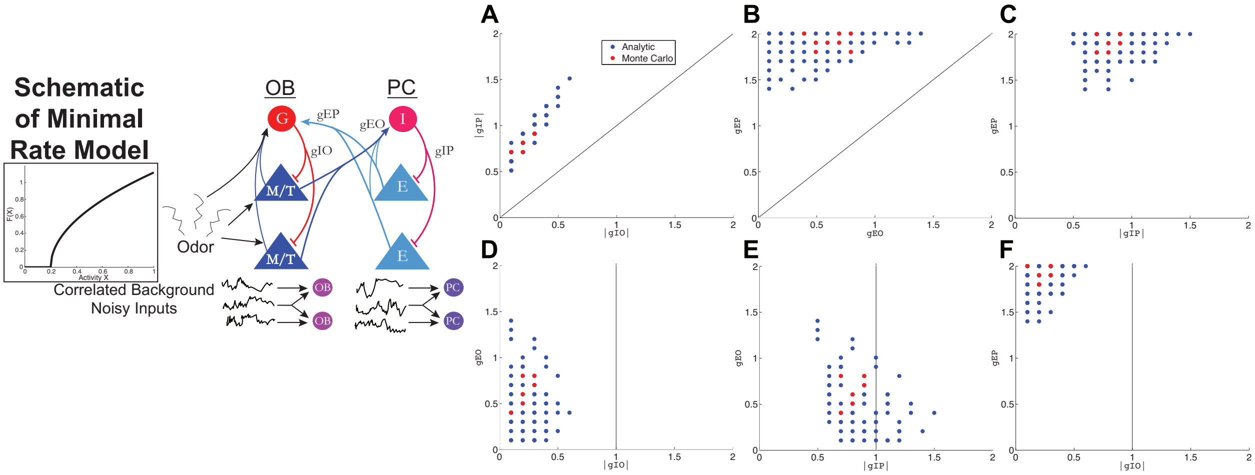

Figure S12: **Analytic approximation results are robust to choice of transfer function.** The results of the fast analytic theory for the rate model using a truncated square root transfer function $F(X) = 1.25\sqrt{X - 0.2}H(X - 0.2)$ are qualitatively similar to the results with the more common sigmoidal function in the main text. Here we have omitted the E to I connections within OB and PC because it does not qualitatively change the results. A: The inhibitory conductance within the PC population $|gIP|$ is larger than in the OB population gOP. B: The excitatory conductance from PC to OB gEP is generally larger than OB to PC gEO. C: Both gEP and $|gIP|$ are relatively large. D: $|gIO|$ is relatively small. E: $|gIP|$ is relatively large. F: Again, $|gIO|$ is relatively small.

Full Spiking Model, μ to PC fixed in Spon./Evoked

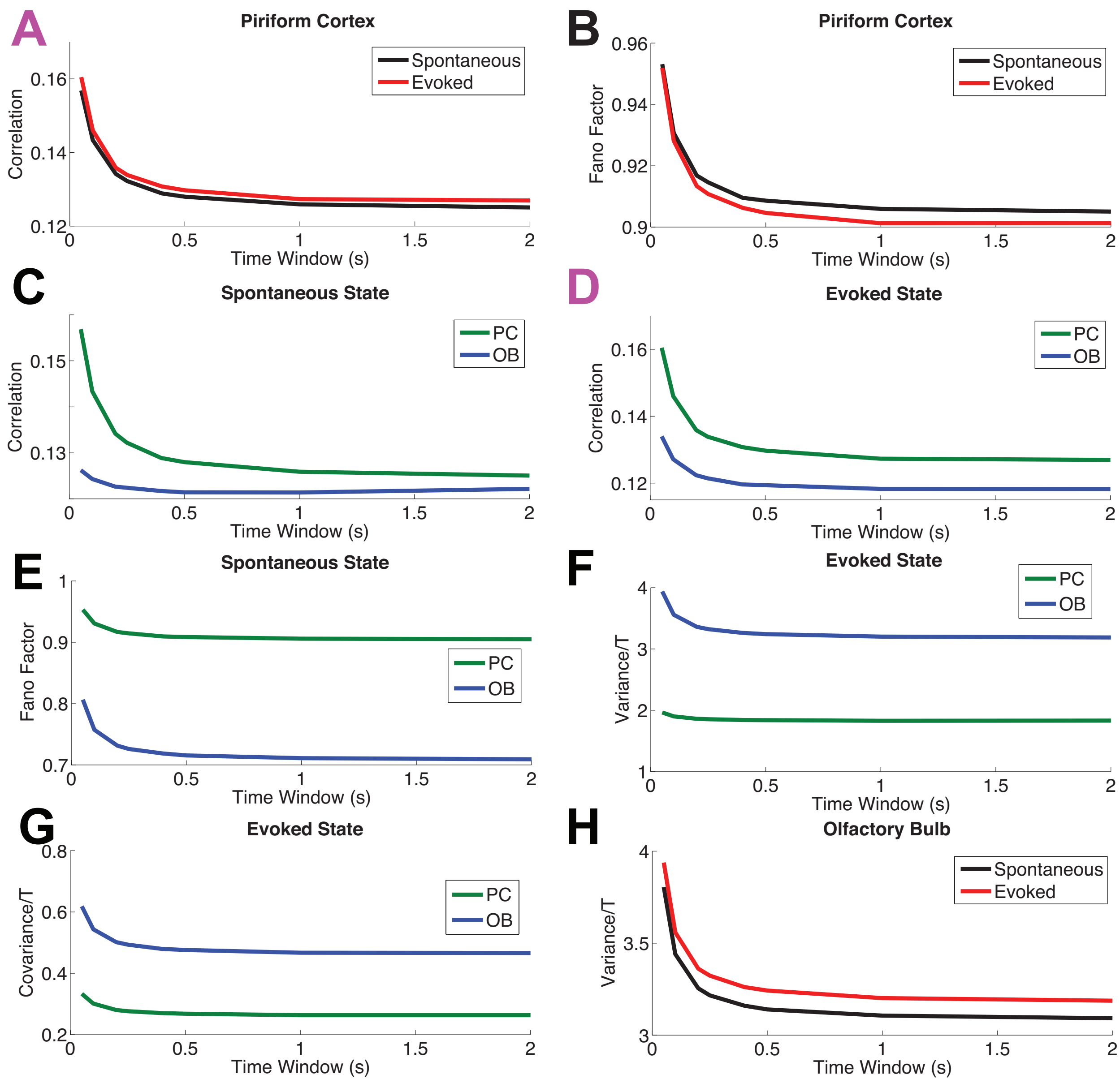

Figure S13: **Mean input to PC must increase in the evoked state.** Showing the results of the full LIF spiking model when the mean input to PC is the same in spontaneous and evoked states: $\mu_{PC} = 0$. The rest of the parameters are the same as in Figure 6 (see main text). The firing rates are: $\nu_{OB}^{Sp} = 5.5 \pm 4.6$, $\nu_{OB}^{Ev} = 5.7 \pm 4.6$, $\nu_{PC}^{Sp} = 2.096 \pm 2.6$, and $\nu_{PC}^{Ev} = 2.13 \pm 2.6$, which barely satisfies the constraint from the experimental data that $\nu_{PC}^{Ev} > \nu_{PC}^{Sp}$. The 8 panels show the constraints on the 2nd order spiking statistics in the same format as in Figure 6 of the main text. The evoked PC correlations decrease but not enough; panels A and D with magenta coloring show the 2 constraints that are violated.

Full Spiking Model, Test 1: gIP < gIO

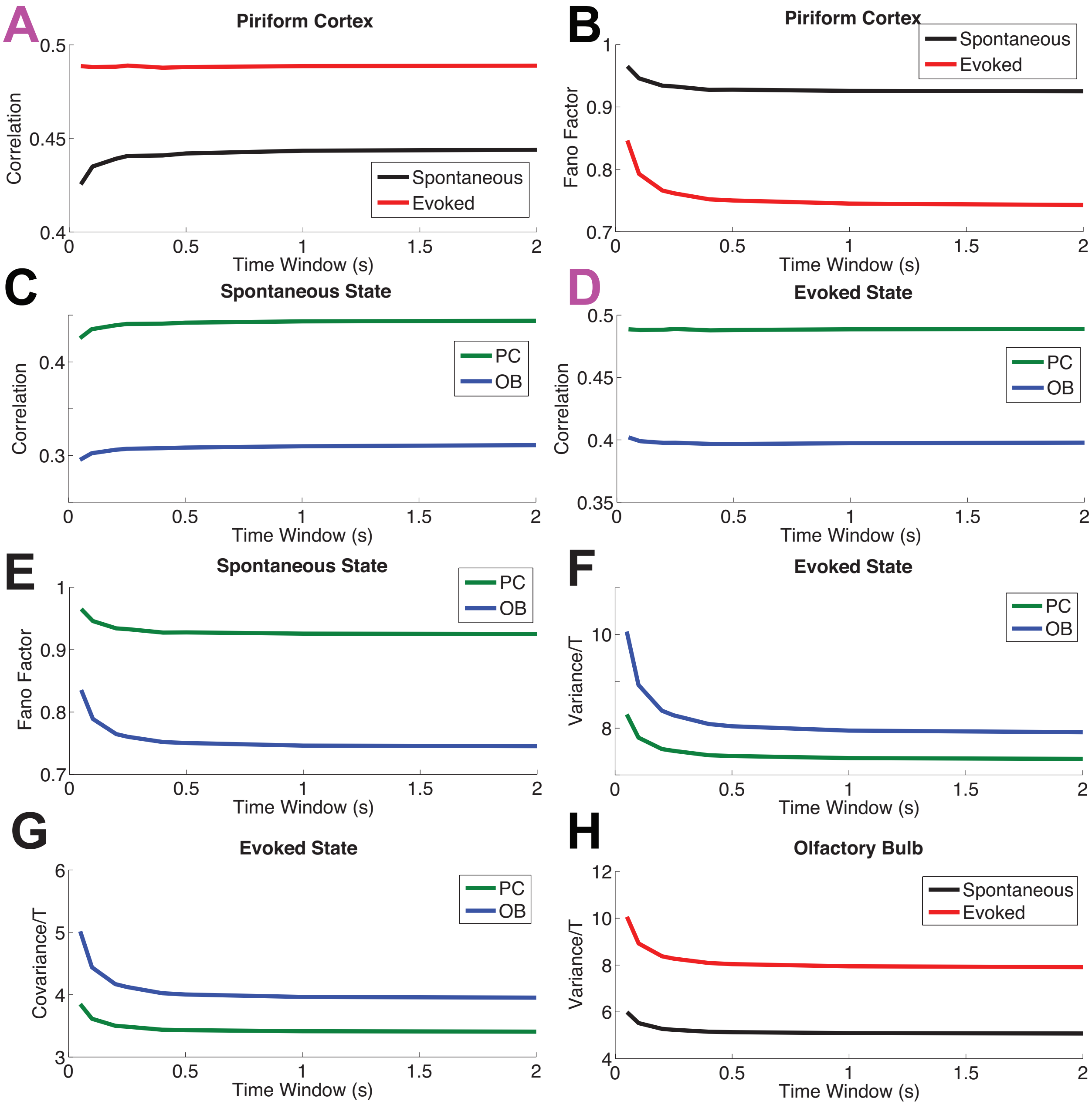

Figure S14: **Violating derived relationship $|gIO| < |gIP|$ results in statistics that are inconsistent with experimental observations.** Showing the results of the full LIF spiking model when $gIP < gIO$; specifically, we set $gIP = 7$ and $gIO = 20$ and set the values of the rest of the parameters to those used in Figure xxx (see main text). The firing rates are: $\nu_{OB}^{Sp} = 7.82 \pm 5.64$, $\nu_{OB}^{Ev} = 13.42 \pm 8.36$, $\nu_{PC}^{Sp} = 3.8 \pm 2.82$, and $\nu_{PC}^{Ev} = 9.67 \pm 6.36$. The 8 panels show the constraints on the 2nd order spiking statistics in the same format as in Figure xxx of the main text. Two constraints are violated; the panels with magenta letters (i.e., A, D) are constraints that are violated.

Full Spiking Model, Test 2: gEP<gEO

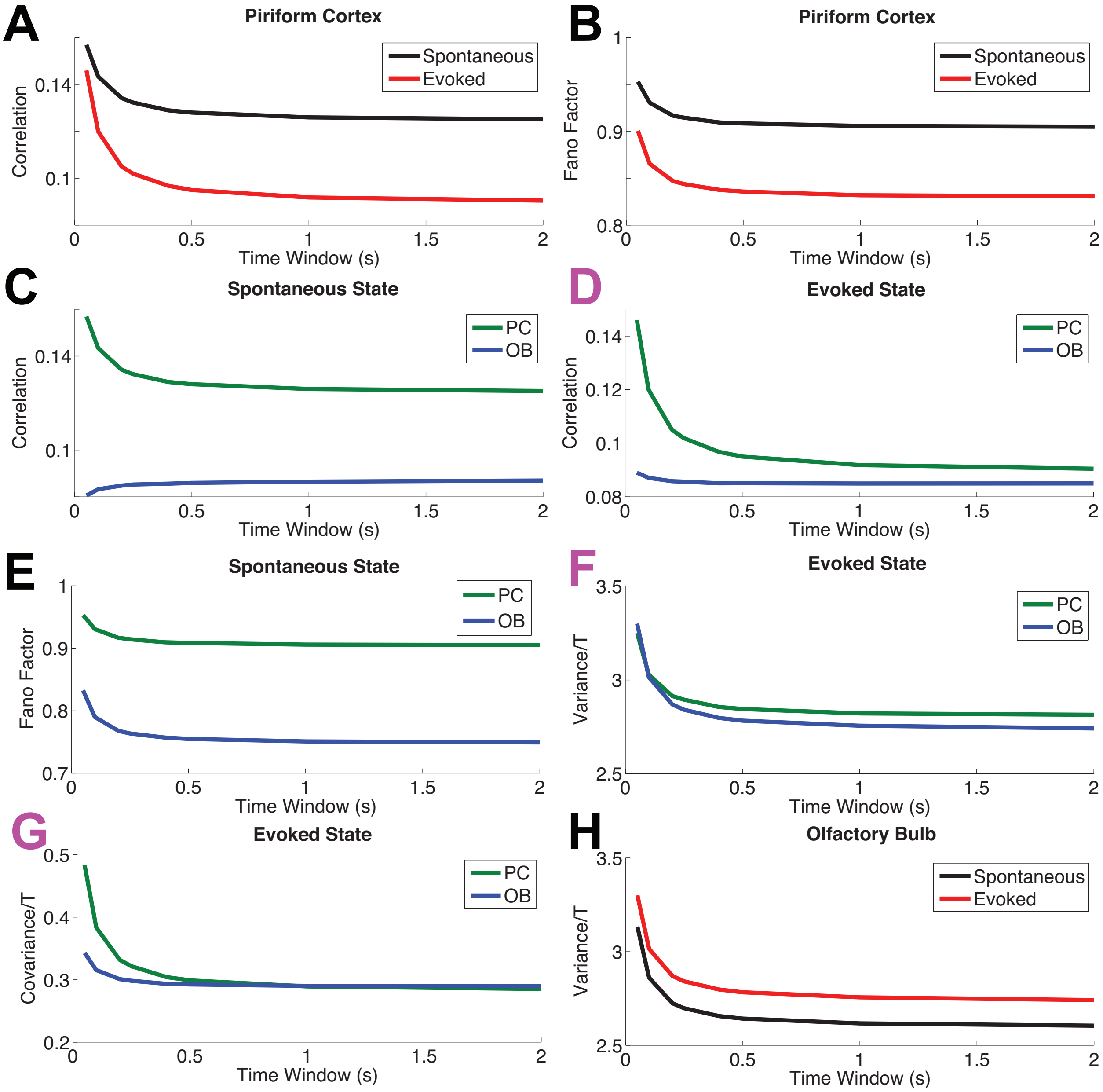

Figure S15: **Violating derived relationship $gEP > gEO$ results in statistics that are inconsistent with experimental observations.** Showing the results of the full LIF spiking model when $gEP < gEO$; specifically, we set $gEP = 1$ and $gEO = 15$; we set the values of the rest of the parameters to those used in Figure 6 (see main text). The firing rates are: $\nu_{OB}^{Sp} = 4.42 \pm 4.09$, $\nu_{OB}^{Ev} = 4.63 \pm 4.01$, $\nu_{PC}^{Sp} = 2.1 \pm 2.64$, and $\nu_{PC}^{Ev} = 4.17 \pm 5.81$. The 8 panels show the constraints on the 2nd order spiking statistics. Three constraints are violated (D, F, G in magenta); note that in G the constraints are violated for small time windows and almost indistinguishable for large time windows.

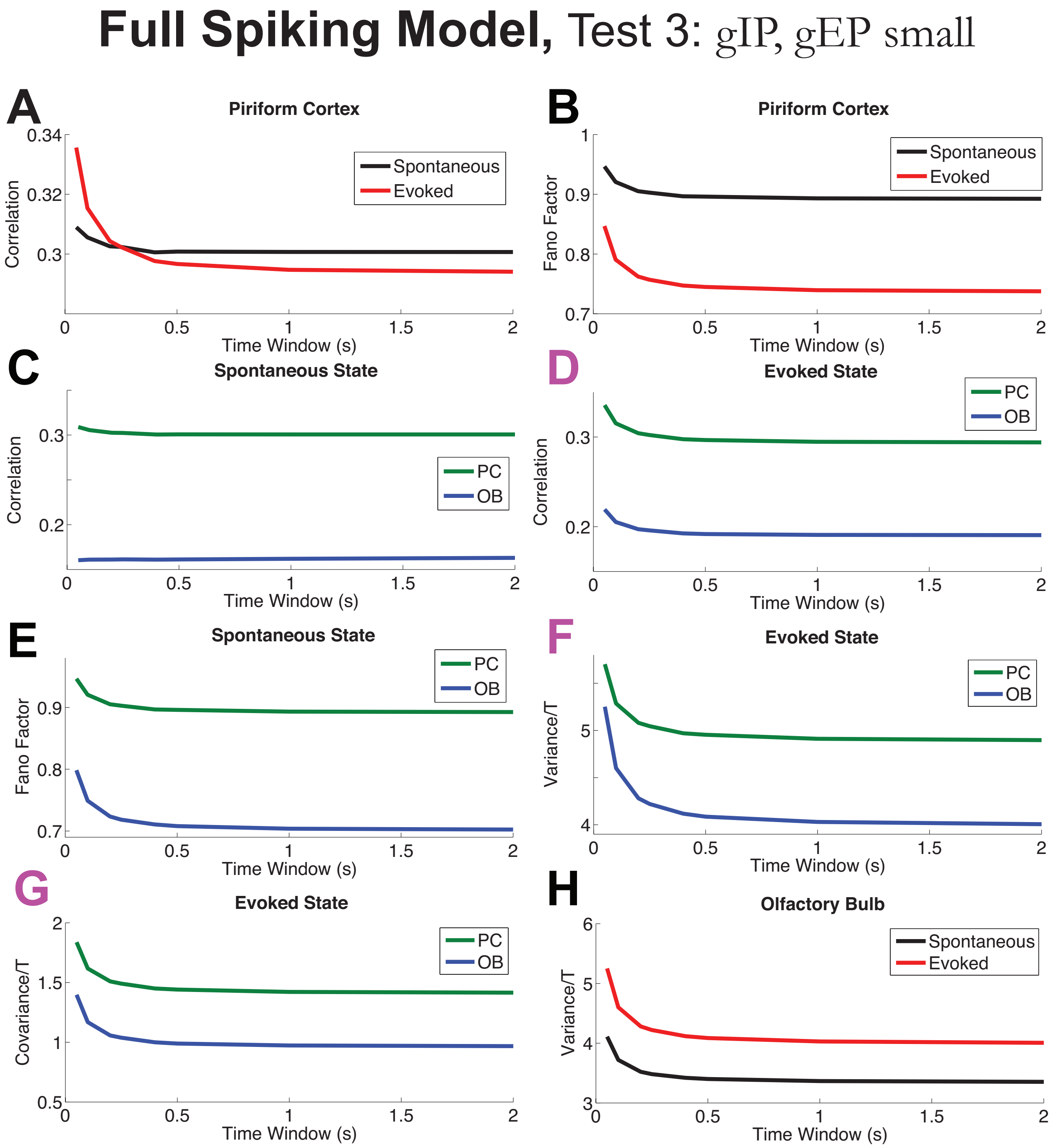

Figure S16: **Violating derived relationship $gEP, gIP \gg gEO, gIO$ results in statistics that are inconsistent with experimental observations.**Showing the results of the full LIF spiking model when gEP and gIP are both relatively small; specifically, we set $gEP = 10$ and $gIP = 10$ and set the values of the rest of the parameters to those used in Figure 6 (see main text). The firing rates are: $\nu_{OB}^{Sp} = 5.98 \pm 4.85$, $\nu_{OB}^{Ev} = 8.17 \pm 5.84$, $\nu_{PC}^{Sp} = 3.03 \pm 2.74$, and $\nu_{PC}^{Ev} = 6.95 \pm 6.1$. The 8 panels show the constraints on the 2nd order spiking statistics. The panels with magenta letters (i.e., D, F, G) are constraints that are violated.
