# Supplementary material for: A theoretical framework for analyzing coupled neuronal networks: Application to the olfactory system
Source: PLoS Comput Biol. 2017 Oct 2;13(10):e1005780. doi: 10.1371/journal.pcbi.1005780 (PMC5638622; doi:10.1371/journal.pcbi.1005780)
Supplement: S3 Text — This file contains supplemental modeling results on a generic cortical-cortical coupled network. Contains Figs. S17-S21, and Table S3 (LIF parameters for cortical networks). S17 Fig: Minimal firing rate model to analyze synaptic conductance strengths; S18 Fig: Detailed spiking LIF model confirms the results from analytic rate model; S19 Fig: Violating derived relationship |gI1| < |gI2| results in statistics that are inconsistent with experimental observations; S20 Fig: Violating derived relationship gE2, gI2 ≫ gE1, gI1 results in statistics that are inconsistent with experimental observations; S21 Fig: Violating derived relationship gE2 > gE1 results in statistics that are inconsistent with experimental observations. (PDF) [file pcbi.1005780.s003.pdf]

Supplementary Material: Cortical-Cortical Network

Andrea K. Barreiro[#] ; Shree Hari Gautam[‡] ; Woodrow L. Shew[‡] ; Cheng Ly[†]

# Department of Mathematics, Southern Methodist University, Dallas, TX 75275 U.S.A.
‡Department of Physics, University of Arkansas, Fayetteville, AR 72701 U.S.A.
†Department of Statistical Sciences and Operations Research, Virginia Commonwealth University, Richmond, VA 23284 U.S.A.
∗ E-mail: abarreiro@smu.edu ; shgautam@uark.edu; woodrowshew@gmail.com ; CLy@vcu.edu

This text shows that the theoretical framework can be applied to a canonical cortical-cortical strongly coupled region. We use the same experimental data constraints from our simultaneous dual-array recordings in the olfactory bulb and piriform cortex, but note that the anatomical connections are **not** the ones described here.

Minimal Firing Rate Model

The minimal firing rate model results in Fig S17 have the same parameters and configuration as in the main text except the E to I connections within a region are omitted. The derived relationships are qualitatively the same as in the main text:

$$|gI1| < gE1 < gE2 \lesssim |gI2|.$$

Leaky Integrate-and-Fire Model of the generic Cortical–Cortical Circuit

We use a generic spiking neural network model of leaky integrate-and-fire neurons to test the results of the theory again. The following model is very much like the LIF model in the main text, with the main differences being in the network connection strengths and the size of C1 (60 here instead of 100 for OB in the main text). There were $N_{C1} = 60$ total C1 cells, of which we set 80% (48) to be excitatory and 20% (12) inhibitory. The equations for the C1 cells are, indexed by

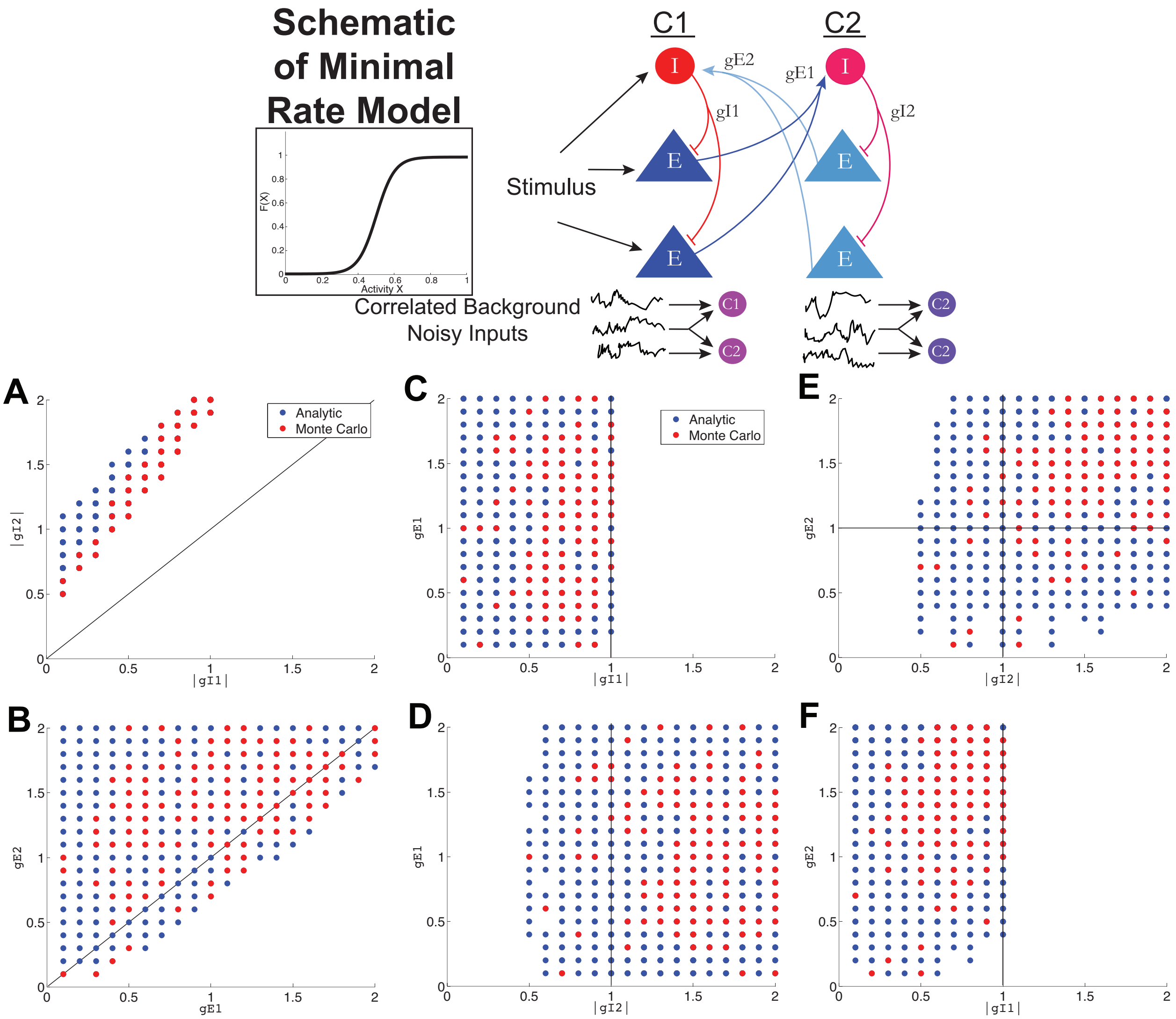

Figure S17: **Minimal firing rate model to analyze synaptic conductance strengths.** This firing rate model only incorporates a subset of the conductances. Each plot shows parameter sets that satisfy all 12 data constraints in Table 1 (main text, substitute OB with C1, PC with C2), projected into a two-dimensional plane in parameter space. The blue dots show the result of the fast analytic method that satisfy all constraints; the red dots show the Monte Carlo simulations that satisfy all 12 constraints. For computational purposes, we only tested the Monte Carlo on parameter sets that first satisfied the constraints in the fast analytic method. (A) The magnitude of the inhibition within C2 ($|gI2|$) is greater than the magnitude of the inhibition within C1 ($|gI1|$); all dots are above the diagonal line. (B) The excitation from C2 to C1 ($gE2$) is generally (but not always) larger than the excitation from C1 to C2 ($gE1$). (C) The inhibition within C1 is generally weak; dots are to the left of the vertical line. (D) The inhibition within C2 is generally strong; dots are to the right of the vertical line. (E) Shows again that excitation from C2 and inhibition within C2 are both strong. (F) Shows again that excitation from C1 to C2 is relatively small. See Table 3 (main text) for parameter values.

$k \in \{1, 2, \ldots, N_{C1}\}$:

$$
\begin{aligned}
\tau_m \frac{dv_k}{dt} &= \mu_{C1} - v_k - g_{k,XI}(t)(v_k - \mathcal{E}_I) - g_{k,XE}(t)(v_k - \mathcal{E}_E) \\
&\quad -g_{k,XC2}(t - \tau_{\Delta,C2})(v_k - \mathcal{E}_E) + \sigma_{C1}\left(\sqrt{1-\tilde{c}_{C1}}\eta_k(t) + \sqrt{\tilde{c}_{C1}}\xi_o(t)\right) \\
v_k(t^*) &\geq \theta_k \Rightarrow v_k(t^* + \tau_{ref}) = 0 \\
g_{k,XE}(t) &= \frac{\gamma_{XE}}{p_{XE}\,(0.8N_{C1})} \sum_{k' \in \{\text{ presyn C1 E-cells}\}} G_{k'}(t) \\
g_{k,XI}(t) &= \frac{\gamma_{XI}}{p_{XI}\,(0.2N_{C1})} \sum_{k' \in \{\text{presyn C1 I-cells}\}} G_{k'}(t) \\
g_{k,XC2}(t) &= \frac{\gamma_{X,C2}}{p_{X,C2}\,(0.8N_{C2})} \sum_{j' \in \{\text{presyn C2 E-cells}\}} G_{j'}(t) \\
\tau_{d,X} \frac{dG_k}{dt} &= -G_k + A_k \\
\tau_{r,X} \frac{dA_k}{dt} &= -A_k + \tau_{r,X}\alpha_X \sum_l \delta(t - t_{k,l}). \qquad (1)
\end{aligned}
$$

The conductance values in the first equation $g_{k,XI}$, $g_{k,XE}$, and $g_{k,XC2}$ depend on the type of neuron v_k ($X \in \{E, I\}$). The last conductance, $g_{X,C2}(t - \tau_{\Delta,C2})(v_k - \mathcal{E}_E)$, models the excitatory presynaptic input (feedback) from the C2 cells with a time delay of $\tau_{\Delta,C2}$. The conductance variables $g_{k,XY}(t)$ are dimensionless because this model was derived from scaling the original (raw) conductance variables by the leak conductance with the same dimension. The leak, inhibitory and excitatory reversal potentials are 0, $\mathcal{E}_I$, and $\mathcal{E}_E$, respectively with $\mathcal{E}_I < 0 < \mathcal{E}_E$ (the voltage is scaled to be dimensionless, see Table S3). $\xi_k(t)$ are uncorrelated white noise processes and $\xi_o(t)$ is the common noise term to all N_{C1} cells.

The second equation describes the refractory period at spike time t^*: when the neuron's voltage crosses threshold θ_j (see below for distribution of thresholds), the neuron goes into a refractory period for τ_{ref}, after which we set the neuron's voltage to 0.

The parameter γ_{XY} gives the relative weight of a connection from neuron type Y to neuron type X; the parameter p_{XY} is probability that any such connection exists ($X, Y \in \{E, I\}$). G_k is the synaptic variable associated with each cell, and dependent only on that cell's spike times; its dynamics are given by the final two equations in Eq 1 and depend on whether $k \in \{E, I\}$.

Finally, two of the parameters above can be equated with coupling parameters in the reduced model:

$$gE2 = \gamma_{I,C2}; \quad gI1 = \gamma_{EI} \qquad (2)$$

which are dimensionless scale factors for the synaptic conductances.

The C2 cells had similar functional form but with different parameters (see Table S3 for parameter values). We modeled $N_{C2} = 100$ total C2 cells, of which 80% were excitatory and 20%

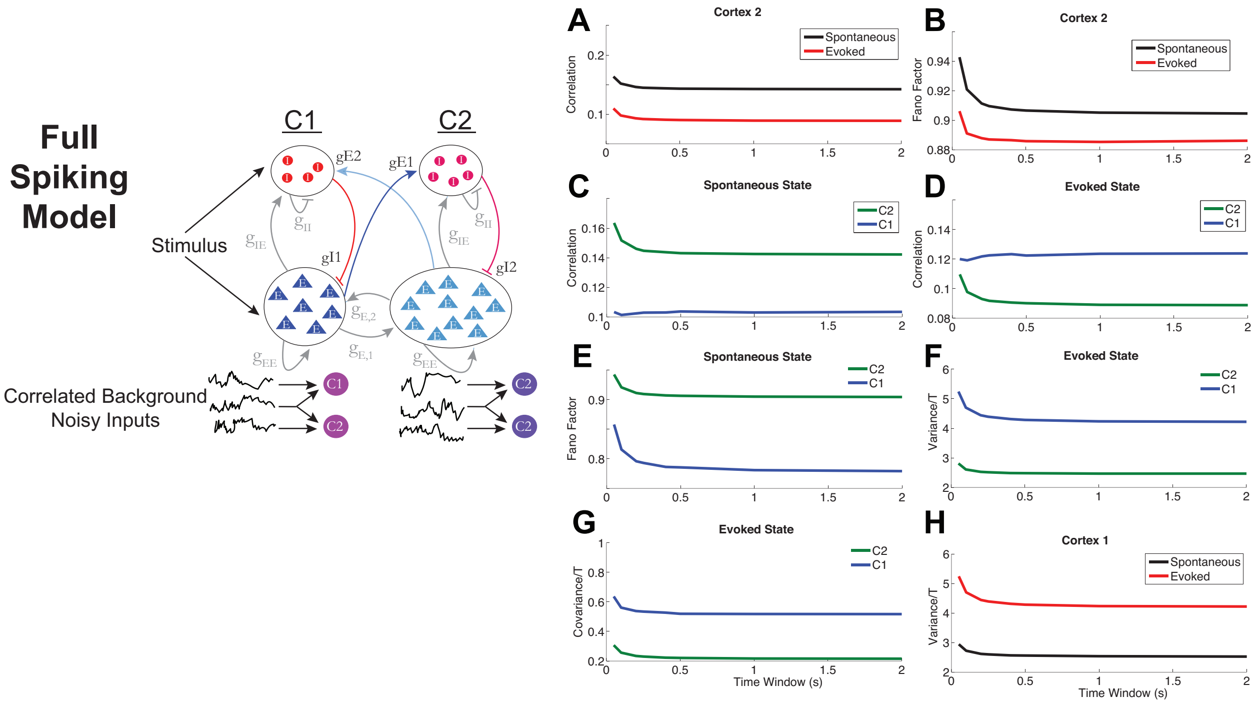

Figure S18: **Detailed spiking LIF model confirms the results from analytic rate model.** Schematic of the LIF model with 2 sets of recurrently coupled E and I cells. There are 12 types of synaptic connections. (A) Pairwise correlations in C2, spontaneous vs. evoked: $\rho_{C2}^{Sp} > \rho_{C2}^{Ev}$. (B) Variability (Fano factor) in C2, spontaneous vs evoked: $FF_{C2}^{Sp} > FF_{C2}^{Ev}$. (C) Correlations in the spontaneous state, C2 vs. C1: $\rho_{C2}^{Sp} > \rho_{C1}^{Sp}$. (D) Correlations in the evoked state, C2 vs. C1: $\rho_{C2}^{Ev} < \rho_{C1}^{Ev}$. (E) Variability (Fano factor) in the spontaneous state, C2 vs. C1: $FF_{C2}^{Sp} > FF_{C1}^{Sp}$. (F) Variability (Fano factor) in the evoked state, C2 vs. C1: $Var_{C2}^{Ev} < Var_{C1}^{Ev}$ in evoked state. (G) Covariances in the evoked state, C2 vs. C1: $Cov_{C2}^{Ev} < Cov_{C1}^{Ev}$. (H) Variability (spike count variance) in C1, spontaneous vs. evoked: $Var_{C1}^{Sp} < Var_{C1}^{Ev}$. The curves show the average statistics over all $N_{C1/C2}$ cells or over all possible pairs $N_{C1/C2}(N_{C1/C2}-1)/2$. We set $gI1 = 7$, $gE1 = 10$, $gI2 = 20$, $gE2 = 15$. See text for model details, and Table S3 for parameter values.

Table S3: **Fixed parameters for the LIF Cortical–Cortical model.**

**Same for both C1 and C2**										
**Parameter**	τ_m	τ_{ref}	$\mathcal{E}_I$	$\mathcal{E}_E$	$\tau_{d,I}$	$\tau_{r,I}$	$\tau_{d,E}$	$\tau_{r,E}$	α_I	α_E
	20 ms	2 ms	-2.5	6.5	10 ms	2 ms	5 ms	1 ms	2 Hz	1 Hz
**Parameter**	N	Spont. μ	Evoked μ	σ	$\tilde{c}$	γ_{EE}	γ_{IE}	γ_{II}	$\gamma_{E,C2/C1}$	$\tau_{\Delta,C2/C1}$
**C1**	60	0.6	0.9	0.05	0.5	2	4	6	1	10 ms
**C2**	100	0	0.4	0.1	0.8	2	4	6	1	5 ms

See Eqs 1–3. All 12 probabilities of connections are set to $p_{XY} = 0.30$ and were randomly chosen (Erdős-Rényi graphs). The synaptic time delay from C1 to C2 is $\tau_{\Delta,C1} = 10$ ms, and from C2 to C1 is $\tau_{\Delta,C2} = 5$ ms. The scaled voltages from mV is: (V+Vreset)/(Vth+Vreset), corresponding for example to Vreset=Vleak=-65 mV, Vth=-55 mV (on average), excitatory reversal potential of 0 mV and inhibitory reversal potential of -90 mV.

inhibitory. The equations, indexed by $j \in \{1, 2, \ldots, N_{C2}\}$ are:

$$\begin{aligned}
\tau_m \frac{dv_j}{dt} &= \mu_{C2} - v_j - g_{j,XI}(t)(v_j - \mathcal{E}_I) - g_{j,XE}(t)(v_j - \mathcal{E}_E) \\
&\quad - g_{j,XC1}(t - \tau_{\Delta,C1})(v_j - \mathcal{E}_E) + \sigma_{C2}\left(\sqrt{1-\tilde{c}_{C2}}\eta_j(t) + \sqrt{\tilde{c}_{C2}}\xi_p(t)\right) \\
v_j(t^*) &\geq \theta_j \Rightarrow v_j(t^* + \tau_{ref}) = 0 \\
g_{j,XE}(t) &= \frac{\gamma_{XE}}{p_{XE}\,(0.8N_{C2})} \sum_{j' \in \{\text{presyn C2 E-cells}\}} G_{j'}(t) \\
g_{j,XI}(t) &= \frac{\gamma_{XI}}{p_{XI}\,(0.2N_{C2})} \sum_{j' \in \{\text{presyn C2 I-cells}\}} G_{j'}(t) \\
g_{j,XC1}(t) &= \frac{\gamma_{X,C1}}{p_{X,C1}\,(0.8N_{C1})} \sum_{k' \in \{\text{presyn C1 E-cells}\}} G_{k'}(t) \\
\tau_{d,X} \frac{dG_j}{dt} &= -G_j + A_j \\
\tau_{r,X} \frac{dA_j}{dt} &= -A_j + \tau_{r,X}\alpha_X \sum_l \delta(t - t_{j,l}).
\end{aligned} \tag{3}$$

Excitatory synaptic input from the C1 cells along the lateral olfactory tract is modeled by: $g_{X,C1}(t - \tau_{\Delta,C1})(v_j - \mathcal{E}_E)$. The common noise term for the C2 cells $\xi_p(t)$ is independent of the common noise term for the C1 cells $\xi_o(t)$. Two of the parameters above can be equated with coupling parameters in the reduced model:

$$gE1 = \gamma_{I,C1}; \quad gI2 = \gamma_{EI} \tag{4}$$

The values of the parameters that were not stated in Table S3 were varied:

$$gI1, \qquad gE1, \qquad gI2, \qquad gE2.$$

To model two activity states, we allowed mean inputs to vary (see Table S3). In contrast to the reduced model, we increased both inputs to C2 cells (from $\mu_{C2} = 0$ in the spontaneous state to $\mu_{C2} = 0.4$ in the evoked state) as well as to C1 cells (from $\mu_{C1} = 0.6$ in the spontaneous state to $\mu_{C1} = 0.9$ in the evoked state).

Finally, we model heterogeneity by setting the threshold values θ_j in the following way. Both C1 and C2 cells had the following distributions for θ_j:

$$\theta_j \sim e^{\mathcal{N}} \tag{5}$$

where $\mathcal{N}$ is normal distribution with mean $-\sigma_\theta^2/2$ and standard deviation σ_θ, so that $\{\theta_j\}$ has a log-normal distribution with mean 1 and variance: $e^{\sigma_\theta^2} - 1$. We set $\sigma_\theta = 0.1$, which results in firing rates ranges seen in the experimental data. Since the number of cells are modest with regards to sampling ($N_{C1} = 60$, $N_{C2} = 100$), we evenly sampled the log-normal distribution from the 5th to 95th percentiles (inclusive).

Violating Derived Relationships Between Conductance Strengths

Similar to the main text, we demonstrate here that violating the relationships derived in the main text results in a subset of the 12 constraints in the experimental data no longer being satisfied in the full spiking network.

Due to the large amount of computing resources required, we cannot exhaustively explore the parameter space; recall that the purpose of the method we developed in the minimal firing rate model is for faster computation. Instead, we distill results into three tests that are exactly the same as in the main text:

1. Make $gI1 > gI2$ by setting $gI1 = 20$ and $gI2 = 7$.
2. Make $gE1 > gE2$ by setting $gE1 = 15$ and $gE2 = 1$
3. Make $gE2$ and $gI2$ relatively smaller by setting $gE2 = 10$ and $gI2 = 10$

The result of Test 1 is that 7 of the 12 constraints are violated (see Fig S19); most importantly stimulus-induced decorrelation of the C2 cells, which is particularly important in the context of coding, was not present. In addition, the C2 firing rates are larger than the C1 firing rates in both states, the evoked C2 correlation is larger than evoked C1 correlation, the spontaneous C2 Fano Factor is larger than spontaneous C1 Fano Factor, and both the variance and covariance of C2 is larger than C1 in the evoked state (all of which violate the constraints from our data).

The result of Test 3 is that 4 of the 12 constraints are violated (see Fig S20), including again stimulus-induced decorrelation of the C2 cells. The evoked C2 correlation is larger than evoked C1 correlation, and both the variance and covariance of C2 are larger than the corresponding quantities in C1 in the evoked state. Both these two tests (1 and 3) indicate that these two qualitative relationships (stronger effective inhibition within C2 and stronger effective presynaptic inputs from C2) are robust with respect to both the detailed LIF spiking model and the minimal firing rate model.

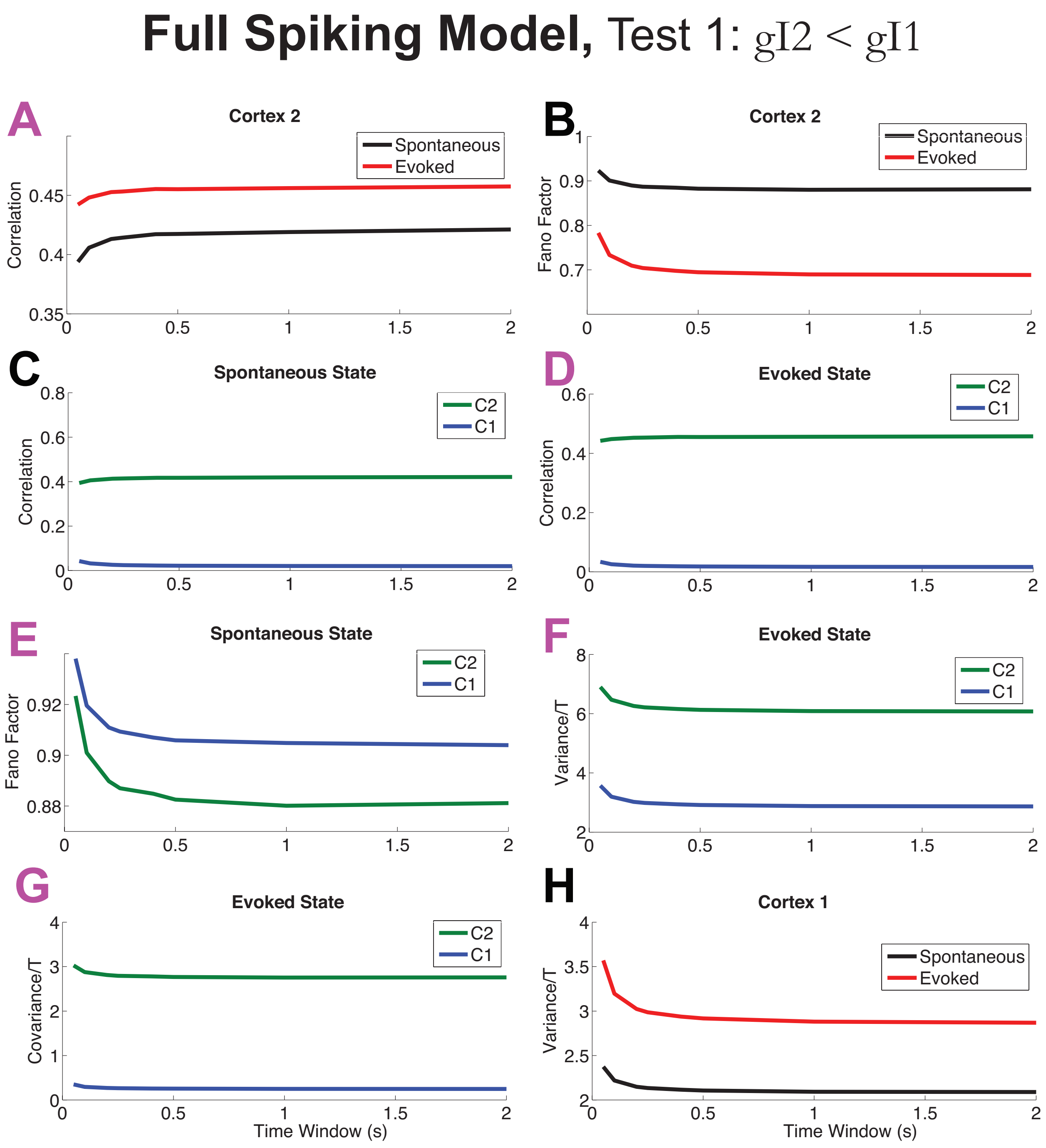

Figure S19: **Violating derived relationship $|gI1| < |gI2|$ results in statistics that are inconsistent with experimental observations.** Showing the results of the full LIF spiking model when $gI2 < gI1$; specifically, we set $gI2 = 7$ and $gI1 = 20$ and set the values of the rest of the parameters to those used previously. The firing rates are: $\nu_{C1}^{Sp} = 2.96 \pm 5$, $\nu_{C1}^{Ev} = 5.94 \pm 11.67$, $\nu_{C2}^{Sp} = 3.43 \pm 1.59$, and $\nu_{C2}^{Ev} = 8.85 \pm 3.38$, which violates the constraint from the experimental data that $\nu_{C1} > \nu_{C2}$ in both states. The 8 panels show the constraints on the 2nd order spiking statistics in the same format as before. The panels with magenta letters (i.e., A, D, E, F, G) are constraints that are violated.

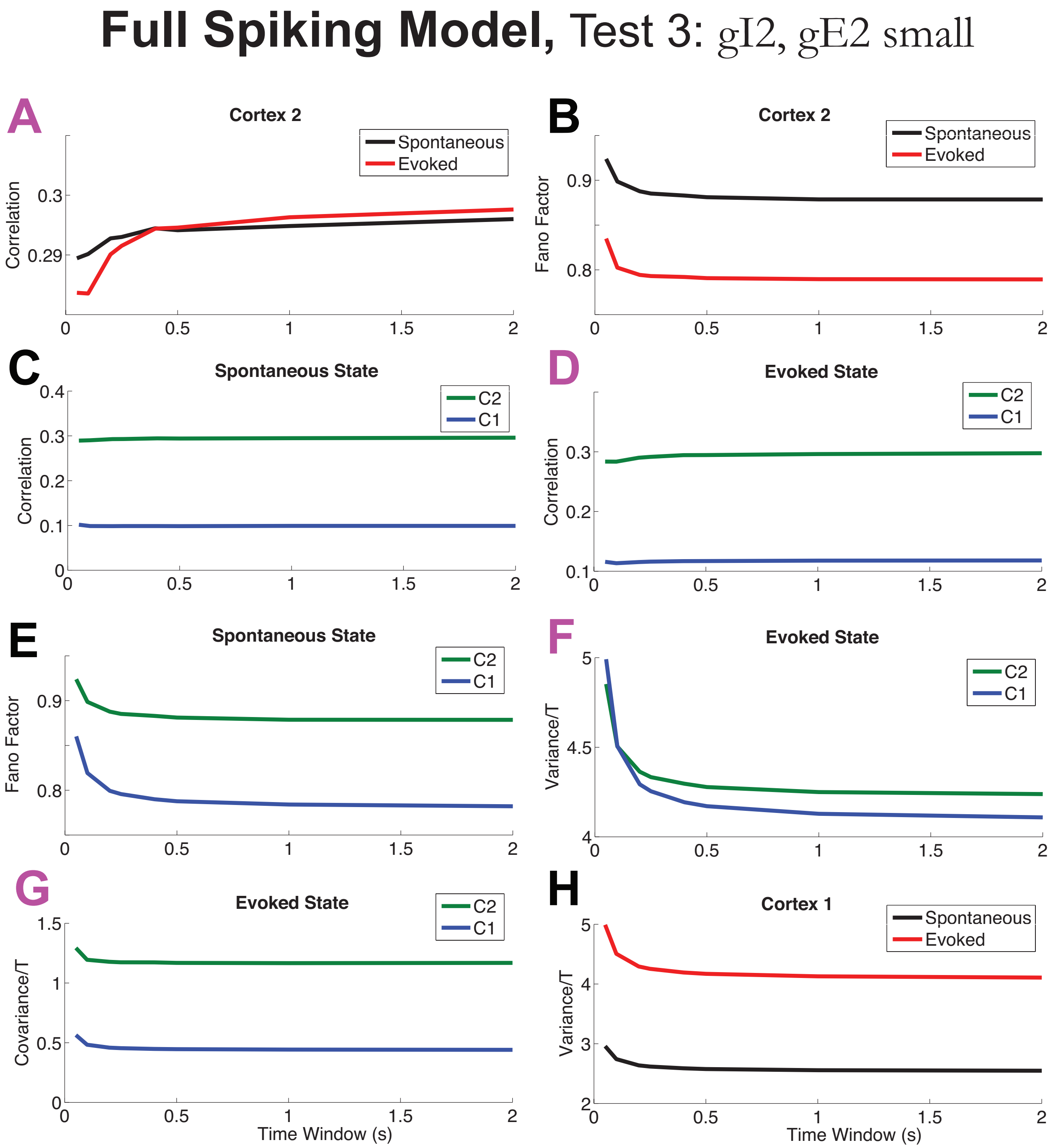

Figure S20: **Violating derived relationship** $gE2, gI2 \gg gE1, gI1$ **results in statistics that are inconsistent with experimental observations.** Showing the results of the full LIF spiking model when $gE2$ and $gI2$ are both relatively small; specifically, we set $gE2 = 10$ and $gI2 = 10$ and set the values of the rest of the parameters to those used in Figure 5 (see main text). The firing rates are: $\nu_{C1}^{Sp} = 3.85 \pm 3.56$, $\nu_{C1}^{Ev} = 8.2 \pm 7.08$, $\nu_{C2}^{Sp} = 2.92 \pm 2.31$, and $\nu_{C2}^{Ev} = 6.45 \pm 6.17$, which violates the constraint from the experimental data that $\nu_{C1} > \nu_{C2}$ in both states. The 8 panels show the constraints on the 2nd order spiking statistics. The panels with magenta letters (i.e., A, D, F, G) are constraints that are violated.

The result of Test 2 is not as straightforward as the others. We did not exhaustively search parameter space due to the vast computational resources this would require, but in several parameter sets with $gE1 > gE2$, we found the resulting network statistics could still satisfy all of the constraints (e.g., with $gE1 = 15$ and $gE2 = 1$, as well as with $gE1 = 20$ and $gE2 = 1$). The reason for this may be that in the two coupled recurrent networks we chose very different $gI1$ and $gI2$ values to begin with (7 and 20, respectively), and would thus require $gE1$ and $gE2$ to be significantly different to counter-balance this. Also, notice in the minimal firing rate model results in Fig 4B that there are a significant number of red dots below the diagonal, indicating that the relationship $gE2 > gE1$ does not have to strictly hold. However, we did find a condition where this test demonstrates the value of the minimal firing rate model; we changed $\tilde{c}_{C1}$ from 0.5 to 0.6 (recall $\tilde{c}_{C2} = 0.8$). (Note that in the minimal firing model that $c_{C1} = 0.3$ and $c_{C2} = 0.35$, relatively close in value.) The result of Test 2 ($gE1 = 15$ and $gE2 = 1$) with $\tilde{c}_{C1} = 0.6$ is that one constraint is violated: ρ^{Sp}_{C2} is no longer less than ρ^{Sp}_{C1} (see Fig S21). This suggests that the relationship that $gE1 > gE2$ is not as robust as the others and can be violated.

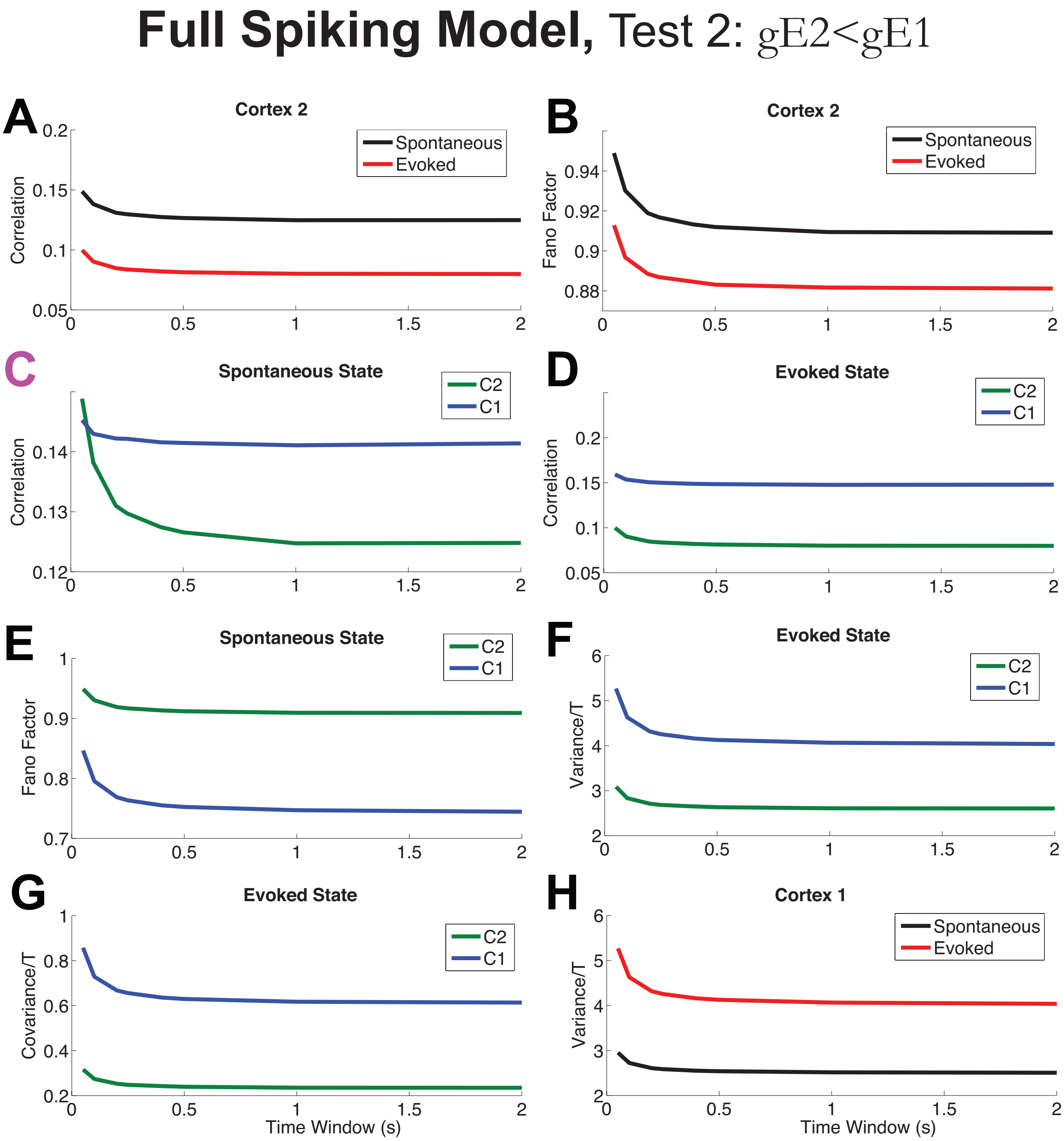

Figure S21: **Violating derived relationship $gE2 > gE1$ results in statistics that are inconsistent with experimental observations.**Showing the results of the full LIF spiking model when $gE2 < gE1$; specifically, we set $gE2 = 1$ and $gE1 = 15$, *and with* $\tilde{c}_{C1} = 0.6$ instead of 0.5; we set the values of the rest of the parameters to those used in Figure 5 (see main text). The firing rates are: $\nu_{C1}^{Sp} = 3.75 \pm 2.61$, $\nu_{C1}^{Ev} = 8.73 \pm 5.12$, $\nu_{C2}^{Sp} = 2.28 \pm 3.32$, and $\nu_{C2}^{Ev} = 4.87 \pm 9.2$. The 8 panels show the constraints on the 2nd order spiking statistics. Only 1 constraint is violated, panel C in magenta.
